# Neurological recovery and antioxidant effect of erythropoietin for spinal cord injury: A systematic review and meta-analysis

**DOI:** 10.3389/fneur.2022.925696

**Published:** 2022-07-19

**Authors:** Ya-yun Zhang, Min Yao, Ke Zhu, Rui-rui Xue, Jin-hai Xu, Xue-jun Cui, Wen Mo

**Affiliations:** ^1^Department of Orthopaedics, LongHua Hospital, Shanghai University of Traditional Chinese Medicine, Shanghai, China; ^2^Department of Orthopaedics, Spine Disease Institute, LongHua Hospital, Shanghai University of Traditional Chinese Medicine, Shanghai, China

**Keywords:** erythropoietin, spinal cord injury, neurological recovery, antioxidant, meta-analysis

## Abstract

**Background:**

To critically evaluate the neurological recovery effects and antioxidant effects of erythropoietin (EPO) in rat models of spinal cord injury (SCI).

**Methods:**

The PubMed, EMBASE, MEDLINE, ScienceDirect, and Web of Science were searched for animal experiments applying EPO to treat SCI to January 2022. We included studies which examined neurological function by the Basso, Beattie, and Bresnahan (BBB) scale, as well as cavity area and spared area, and determining the molecular-biological analysis of antioxidative effects by malondialdehyde (MDA) levels in spinal cord tissues. Meta-analysis were performed with Review Manager 5.4 software.

**Results:**

A total of 33 studies were included in this review. The results of the meta-analysis showed that SCI rats receiving EPO therapy showed a significant locomotor function recovery after 14 days compared with control, then the superiority of EPO therapy maintained to 28 days from BBB scale. Compared with the control group, the cavity area was reduced [4 studies, weighted mean difference (WMD) = −16.65, 95% CI (−30.74 to −2.55), *P* = 0.02] and spared area was increased [3 studies, WMD =11.53, 95% CI (1.34 to 21.72), *P* = 0.03] by EPO. Meanwhile, MDA levels [2 studies, WMD = −0.63 (−1.09 to −0.18), *P* = 0.007] were improved in the EPO treatment group compared with control, which indicated its antioxidant effect. The subgroup analysis recommended 5,000 UI/kg is the most effective dose [WMD = 4.05 (2.23, 5.88), *P* < 0.0001], although its effect was not statistically different from that of 1,000 UI/kg. Meanwhile, the different rat strains (Sprague-Dawley vs. Wistar), and models of animals, as well as administration method (single or multiple administration) of EPO did not affect the neuroprotective effect of EPO for SCI.

**Conclusions:**

This systematic review indicated that EPO can promote the recovery of the locomotor function of SCI rats. The mechanism exploration of EPO needs to be verified by experiments, and then carefully designed randomized controlled trials are needed to explore its neural recovery effects.

## Introduction

Spinal cord injury (SCI) is an injury to the spinal cord caused by trauma or disease, which may lead to alterations to the normal motor, sensory or autonomic function of the spinal cord (1, 2). Traumatic SCI is a disastrous event associated with high morbidity and mortality (3). With the development of the modern transportation and construction industries, incidence of SCI has sharply risen up (4, 5). The incidence of SCI is ~180,000 cases per year worldwide (6). Half of the people with SCI have a huge impact on their daily lives, such as limb paralysis and urine obstacles (7). In the last 20 years, drug therapy, Chinese herbal medicine, and stem cell transplantation have been studied widely across the world and have been the focus of a significant effort.

At present, the main methods for the therapy of SCI include surgical treatment, drug treatment, hyperbaric oxygen therapy, and physical therapy. During the SCI window, the main strategies used to restrict secondary damage are surgical decompression, therapeutic hypothermia, and the administration of high doses of glucocorticoids (8, 9). However, there may be an increased risk of gastrointestinal hemorrhage and respiratory tract infection after high-dose methylprednisolone treatment early after SCI (10). Surgical treatment of patients with spinal cord injury is not satisfactory, and it is usually related to poor prognosis (11). At present, the drugs studied include resveratrol, Gangliosides, quercetin, and mesenchymal stem cells, which have certain effects on the treatment of SCI, but there are still limitations (12–15).

Erythropoietin (EPO), an evolutionarily conserved hormone specially produced within the kidney, has been properly documented for its quintessential role in erythropoiesis. EPO belongs to the type 1 cytokine superfamily and has 165 amino acids forming four α helices 1 (16). EPO binds to a homodimeric EPO receptor, which induces phosphorylation of numerous tyrosine residues within the intracellular domain of the receptor, which ultimately ends in the activation of prosurvival, proliferation, and prodifferentiation genes in the progenitor cells (17). In recent years, numerous studies have shown that EPO acts far beyond erythropoiesis. In hypoxia, trauma, or inflammation, many tissues produce EPO at the borders surrounding injury sites; EPO plays a central role in tissue protection and restoration. This may include the mechanisms of producing anti-apoptotic factors, limiting oxidative stress, inhibiting the production of nitric oxide, stimulating angiogenesis, reducing glutamate toxicity, and relieving inflammation by activating antioxidant enzymes and inhibiting lipid peroxidation (18–23). However, a few studies have shown that EPO has no positive effect on the recovery of motor function, the reduction of lesion volume, and the increase in the number of axons (24, 25). Moreover, the demonstration of EPO and its analogs' wide neuroprotective results in animal models of cord lesion and traumatic brain injury, and human trials like stroke, ought to inspire scientists and clinicians to design clinical trials assessing the efficacy of these pharmacological compounds on SCI (26–28). Therefore, the neurological recovery and antioxidant effects of EPO for SCI should be evaluated.

Therefore, our aim was to verify the hypothesis that EPO was more effective than placebo on locomotor function recovery in the rat SCI model. A systematic review and analysis was performed to assess the neurological recovery and anti-oxidative results of EPO in SCI rats.

## Data and methods

### Search strategy

Literature retrieval was conducted from English databases, including PubMed, EMBASE, MEDLINE, ScienceDirect, and Web of Science. Relevant studies were found using the following search terms “EPO,” “epoetin,” “erythropoietin,” “Procrit,” “epogen,” “erupt,” “Biopoin,” “Eporatio,” “darbepoetin,” “DARB,” “ARANESP,” “NESP,” “Neorecormon,” “erythropoietin stimulating proteins,” “erythropoietin stimulating agents,” “ESA,” “epoetin alfa,” “darbepoetin alfa,” “spinal cord injury,” “spinal cord injuries,” “traumatic spinal cord injury,” and “spinal cord repair.” In addition, the bibliographies of all included articles and important conference papers were searched for additional relevant studies. Retrieval of literature was carried out from the inception dates of the databases to January 2022. No language restriction was used in the literature search, and the search was limited to studies in rats.

### Study selection

Two reviewers (YYZ and MY) evaluated each article separately for preliminary screening, according to the title and abstract, then read through the full text for secondary screening. Disagreements were resolved by agreement and discussion with a third party (JHX).

### Inclusion and exclusion criteria

Trials were included if they met the following criteria for participants, interventions, comparisons, outcomes, and study design (PICOS) criteria: (1) Participants: Experimental rat studies that involved at any age or gender with traumatic SCI, including contusion, impactor damage, crush and compression injury; (2) Intervention: The dose and the administration method of EPO, duration of treatment, and follow-up time were unrestricted; (3) Comparison: Physiological saline, vehicle, or no treatment were included in the control groups; (4) Outcomes: The Basso, Beattie, and Bresnahan (BBB) score was prioritized for collection as the primary outcome (29). Cavity area and spared area was also used to evaluate the neurological recovery of EPO, as secondary outcomes, as well as malondialdehyde (MDA) to antioxidant effect. Articles containing other outcome measures were also included for the final mechanism summary. (5) Study design: Comparision of EPO with control in SCI rats.

Studies with one or more of the following conditions were excluded: (1) Nontraumatic injury, penetrating injury, and complete transverse spinal cord injury of SCI rat models were excluded. (2) The study of clinical case reports, genetically modified rats, and EPO combined with other intervention treatments was excluded. (3) Review, duplicated, and not related articles were excluded.

### Data extraction

The information was independently extracted from papers by two authors. The following data were extracted: first author, publication year, animal gender, days of rats, weight of rats, number of rats per group, model of SCI, SCI level, type of intervention, timing of intervention, duration of intervention, the daily dose of EPO and outcomes. The mean ± standard deviation (SD) of each outcome was also extracted for pooled analysis. Disagreements were resolved by discussion, and a third reviewer's opinion was asked for when necessary. If the data were missing or incomplete, numerical values were requested from the authors *via* email, or GetData Graph Digitizer 2.24 was used to estimate numerical values from the graphs (http://getdata-graph-digitizer.com/download.php).

### Assessment of risk of bias in included studies

The Collaborative Approach to Meta Analysis and Review of Animal Data From Experimental Stroke (CAMARADES) 10-item checklist were used to assess the quality and design of the studies by two independent investigators (30). The CAMARADES list includes the following: (1) peer-reviewed journal; (2) temperature control; (3) animals were randomly allocated; (4) blind established model; (5) blinded outcome assessment; (6) anesthetics used without marked intrinsic neuroprotective properties; (7) animal model (diabetic, advanced age or hypertensive); (8) calculation of sample size; (9) statement of compliance with animal welfare regulations; (10) possible conflicts of interest.

Two reviewers assessed the risk of bias. Bias was marked as high or low risk, as well as “unclear” indicated that the risk of bias was unclear. The symbol “+” was used to marked low risk, and it was also recorded as the point of quality score. Disagreements were resolved by discussion, and a third reviewer's opinion was asked for when necessary.

### Statistical analysis

Statistical analyses were performed with version 5.4 of the Cochrane Collaboration Review Manager (RevMan). Data from all EPO groups were pooled to compare with the SCI groups; data were pooled if outcomes were reported by at least two studies and continuous variables were expressed as weighted mean difference (WMD) or standardized mean difference (SMD), both with 95% confidence interval (CI). A chi-square-based Q test was used to measure between-study heterogeneity. Statistical significance was defined at *P* < 0.05. *I*^2^ statistic was calculated to quantify the proportion of the total variation across studies due to heterogeneity (31). The fixed effect models were adopted, if the heterogeneity was not obvious (*P* > 0.1; *I*^2^ ≤ 50%); when *P* ≤ 0.1; *I*^2^ > 50%, random effect models were used (32, 33). Subgroup analysis was performed according to different conditions, such as rat strain, modeling method, dosage and time of administration. If we included at least 10 studies in a meta-analysis related to primary outcomes, funnel plots were used to test the potential risk of publication bias (34).

## Results

### Search results

**Figure 1:**
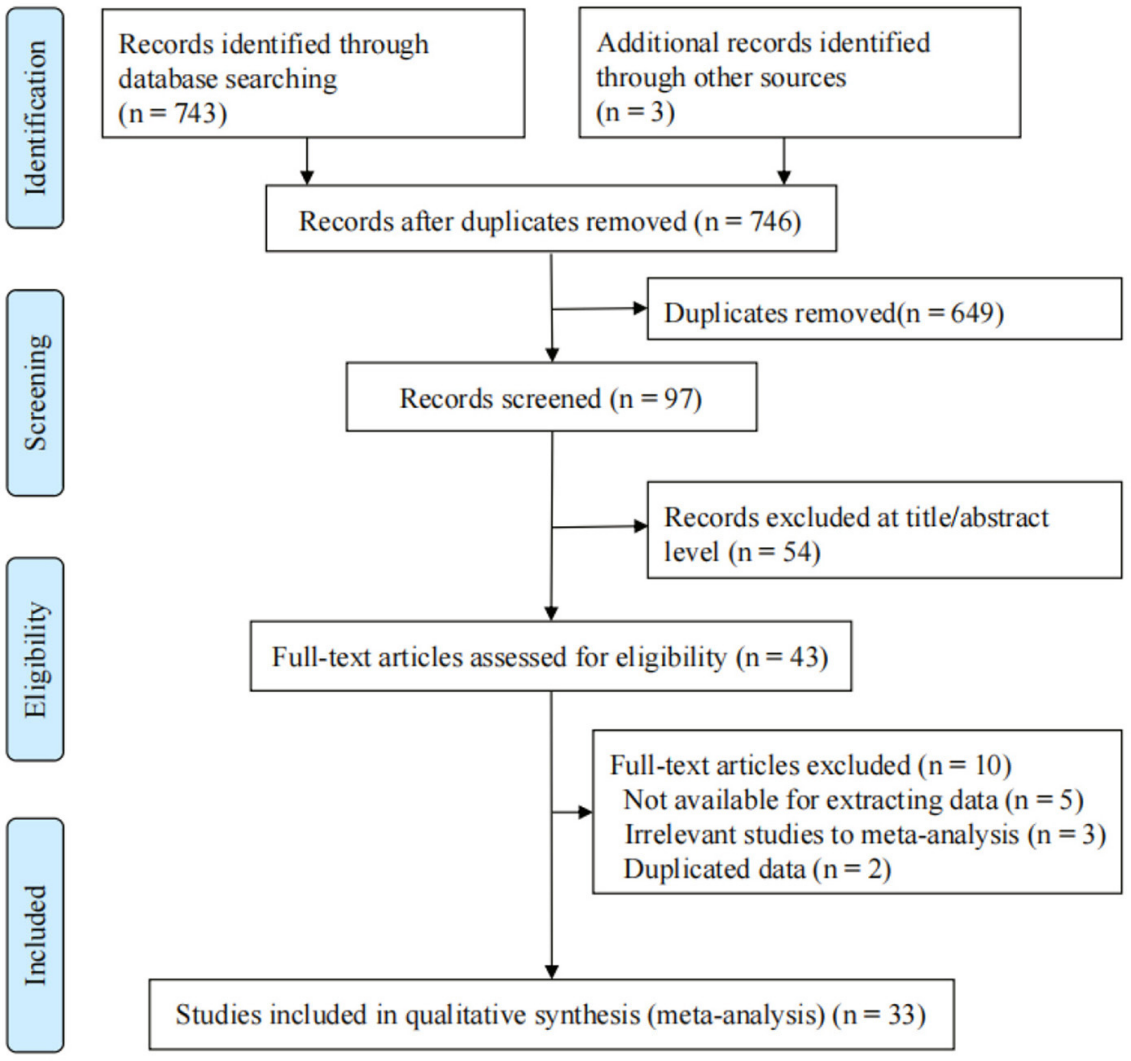
PRISMA 2009 Flow diagram.

Among the 746 articles found in the initial search strategy, 649 similar and duplicated studies were removed. Fifty-four articles were ruled out by the titles and abstracts; 43 studies were retained. After evaluating the full texts, 10 articles were excluded, 33 independent studies were chosen for the final meta-analysis after evaluation. The flow chart of the study selection is summarized in Figure 1.

### Characteristics of included studies

Characteristics of the studies included in this systematic review are shown in Table 1. Of the 33 articles that met the inclusion criteria, all were published in English. Sprague-Dawley rats were used in 25 studies and Wistar rats were used in eight studies. Nineteen studies used male rats, nine studies were conducted using female rats and the other studies had no gender information. Only two studies had age information (57, 59). Studies used aneursym clip, impactor, aortic occlusion, deep hypothermic circulatory arrest or bulldog clamp to induce SCI in rats.

**Table 1:** Characteristics of the included studies.

**References**	**Animals**	**Model**	**No. of rats**	**Groups**	**Met**	**Neurologic outcome**
Gorio et al. (18) (Italy)	Adult female Wistar rats (180–300 g); Adult female SD rats (240–260 g)	Aneursym Clip: T3, 0.6 N, 1 min; Impactor: T9, 1 mm, 1 N, 1 s	6/14/14/14; 14/14/14/14	Aneursym Clip A. Sham B. SCI C. SCI + rhEPO (1,000 UI/kg) D. SCI + rhEPO (1,000 UI/kg, tid) Impactor A. SCI B. SCI + rhEPO (5,000 UI/kg) C. SCI + rhEPO (5,000 UI/kg, 7 d) D. SCI + rhEPO (500 UI/kg, 7 d)	1,000 UI/kg, *i.p*, bw, tid, after injury, 1 h; 5,000 UI/kg or 500 UI/kg, 7 d, *i.p*, after injury, 1 h	BBB scale, swimming score
Leist et al. (35) (USA)	Rats	Aortic occlusion: ischemia for 60 min, followed by reperfusion	6/6/6	A. SCI + saline B. SCI + EPO C. SCI + CEPO	EPO: 5 g/kg; CEPO: 50 g/kg, *i.v*., after injury, 0, 24, 72 h, 3 times a wk	BBB scale
Gorio et al. (36) (Italy)	Adult SD rats (240–260 g)	Impactor: T9, 1 N, 1 s	18/18/18/18	A. SCI B. SCI + EPO C. SCI + MPSS D. SCI + EPO + MPSS	5,000 UI/kg-bw, *i.p*, after injury, 30 min	BBB scale
Grasso et al. (37) (Italy)	SD rats (275–300 g)	Aneurysm clip: T3, 58 g, 1 min	6/6/6/6/6/6/6/12	A. Sham B. SCI C. SCI + asialoEPO 24 h D. SCI + asialoEPO E. SCI + multiple doses of asialoEPO F. SCI + EPO 24 h G. SCI + EPO H. SCI + EPO multiple doses	10 mg/kg, *i.v*., before injury, 24 h, single doses; after injury, multiple doses	BBB scale
Choi et al. (38) (Korea)	Male SD rats (300–350 g)	Aneurysm clip: T8–10, pressure of 50 gm/cm^2^, 10 min	12/13/13/13	A. SCI B. SCI + pSV-VEGF C. SCI + pEpo-SV-VEGF D. SCI+pRTP801-VEGF	Plasmid DNA, *i.p*, after injury at a rate of 0.5 μl/ml.	BBB scale
Okutan et al. (39) (Turkey)	Adult female Wistar rats (210–250 g)	Impactor: T7-9, 3 mm in diameter, 10 cm, 40 g/cm	8/8/8/8/	A. Sham B. SCI C. SCI + vehicle D. SCI + MPSS E. SCI + EPO	1,000 UI/kg, *i.p*, after injury.	BBB scale
Vitellaro-Zuccarello et al. (40) (Italy)	Adult male SD rats (240–270 g)	Impactor: T9, 2.3 mm in diameter, 1 mm, 1 N for 1 s	7/7/7	A. Sham B. SCI C. SCI + rhEPO	5,000 UI/kg, *i.p*, 30 min after injury	BBB scale
Vitellaro-Zuccarello et al. (41) (Italy)	Adult male SD rats (240–270 g)	Impactor: T9, 2.3 mm in diameter, 1 mm, 1 N, 1 s	14/14/14/3	A. Normal B. Sham C. SCI D.SCI + rhEPO	5,000 UI/kg, *i.p.*, 30 min after injury	BBB scale
Yazihan et al. (42) (Turkey)	Adult Wistar albino rats (200–220 g)	Aneurysm clip: T10, 40 g, 30 s	7/7/7/7/7	A. Sham B. SCI C. SCI + EPO D. SCI + ketamine E. SCI + EPO + ketamine	150 UI/kg, *i.p*. 1/2 h and 1 h following the injury	/
Fumagalli et al. (43) (Italy)	Adult SD rats (240–260 g)	Impactor: T9, 1 N, 1 s	8/8/8/8	A. Sham B. SCI C. SCI + MPSS D. SCI + EPO	1,000 UI/kg, *i.p*. 30 min after injury	BBB scale
Mann et al. (44) (Canada)	Male SD rats (320–340 g)	Impactor: T9-10, 0.2 kdyne, 1.5 mm, 300 m/s	11/12/11	A. SCI B. SCI+EPO C. SCI+darbepoetin	5,000 UI/kg, *i.p*. 1 h after injury	BBB scale, horizontal ladder test to measure footfalls
Pinzon et al. (45) (America)	Adult female Wistar rats (220–280 g)	Aneurysm clip: T3, 20 g, 10 s; Impactor: T9, 12.5 cm, 10 g	10/10/10/10/ 15/15/15/3	Aneurysm clip A. Sham B. SCI + normal saline solution C. SCI + rhEPO1 D. SCI + rhEPO1 24/48 h Impactor A. Sham B. SCI + normal saline solution C. SCI + rhEPO3 D. SCI + rhEPO3 24/48 h	1,000 UI/kg, *i.p.*, in 1 or 3 doses after ischemia; 5,000 UI/kg, *i.p.*, in 7 d or single dose after ischemia	BBB scale
Fang et al. (46) (China)	Female SD rats (230–250 g)	Impactor: T10, 10 g, 50 mm, 20 s	20/20/20	A. Sham B. SCI C. SCI + EPO	1,000 UI/kg, *i.p*, after injury	BBB scale
Kontogeorgakos et al. (47) (Greece)	Female Wistar rats (270–300 g)	Aneurysm clip: T10, 0.7 N, 60 s	10/10/10	A. SCI B. SCI + EPO-L C. SCI + EPO-H	1,000 UI/kg, *i.p*., after injury and 1 d; 1,000 UI/kg, *i.p*., after injury and 1 d, then every second day (until 25 d)	BBB scale
Huang et al. (48) (China)	Male SD rats (210 g)	Impactor: T10, 10 g, 50 mm, 20 s	20/20/20	A. Sham B. SCI C.SCI + EPO	1,000 UI/kg, *i.p*., after ischemia	BBB scale
Hong et al. (49) (China)	Adult male SD rats (180–200 g)	Impactor: T10, 10 g, 50 mm, 20 s	10/10/10	A. Sham B. SCI C. SCI + EPO	1,000 UI/kg, *i.p*., after injury	BBB scale
Hwang et al. (50) (Korea)	Male SD rats (250–300 g)	Aortic occlusion: ischemia for 10.5 min, followed by reperfusion	8/8/8	A. Sham B. SCI C. SCI + EPO	1,000 UI/kg *i.p*., before ischemia, 24 h	Motor deficit index
Hong et al. (51) (China)	Adult male SD rats (200–220 g)	Impactor: T10, 10 g, 50 mm, 20 s	20/20/20	A. Sham B. SCI C. SCI+EPO	1,000 UI/kg, *i.p*., after ischemia	BBB scale
Jin et al. (52) (China)	Male SD rats (250–300 g)	Aneurysm clip: T8-T9, 30 g	16/16/16	A. Sham B. SCI C. SCI + rhEPO	5,000 UI/kg, *i.p*., after injury, 30 min	BBB scale
Freitag et al. (53) (German)	Male SD rats (300–350 g)	Impactor: A. 150 kdyn	9/9	A. SCI B. SCI + EPO	1,000 UI/kg, *i.v*., after injury, 1 h	BBB scale
Yang et al. (54) (China)	Adult male SD rats (220–260 g)	Impactor: 2 cm, T10, 70 g force; 65821 T; for 1 min to induce crush injury	First stage:8/8/8/8 Second stage:6/6/6/6	First stage: A. SCI B. SCI + rhEPO-3000 UI/kg C. SCI + rhEPO-4000 UI/kg D. SCI + rhEPO-5000 UI/kg Second stage: A. SCI B. SCI + rhEPO-3000 UI/kg C. SCI + rhEPO-4000 UI/kg D. SCI + rhEPO-5000 UI/kg	3,000 UI/kg/ 4,000 UI/kg/ 5,000 UI/kg, *i.p.*, after injury, 2 h	BBB scale
Wu et al. (55) (China)	Male SD rats (200–250 g)	Impactor: T7-10, 10 g, 2.5 cm	23/23/23	A. SCI + DMEM/F12 medium B. SCI + NSCs + DMEM/F12 medium C. SCI + hEPO-NSCs + DMEM/F12 medium	20 μl, DMEM/F12 medium, infused into the subarachnoid cavity, after injury, 5 min	BBB scale
PR de Mesquita Coutinho et al. (25) (Brazil)	Adult male Wistar rats (320-340 g)	Impactor: T8-T12, 10 g, 2.5 cm	12/12/12/12/12	A. Sham B. SCI C. SCI + EPO D. SCI + EPO + FK 506 E. SCI + FK 506	1,000 UI/kg *i.p*., after injury, 5 min	BBB scale
Marcon et al. (56) (Brazil)	Male Wistar rats (254–405 g)	Impactor: T10, 10 g, 2.5 cm, 15 s	12/12/12/12/12	A. Sham B. SCI + saline C. SCI + ganglioside G(M1) D. SCI + EPO E. SCI + ganglioside G(M1) + EPO	1,000 UI/kg, *i.p*. after ischemia	BBB scale
Zhao et al. (57) (China)	SD rats (60 d old, 180–200 g)	Impactor: T10, 10 g, 10 cm	15/15/15	A. Sham B. SCI C. SCI + EPO	300 UI/kg, *i.p*., after injury, 1, 3, 5, 7, and 9 d	Tarlov score, Rivlin and Tator score
Kökoglu et al. (58) (Turkey)	Female SD rats (200–250 g)	Aneurysm clip: 1.43 N	7/7/7/7/7	A. Sham B. SCI C. SCI + solvent D. SCI + EPO E. SCI + tadalafil	2,000 UI/kg, *i.p*, after injury	/
Li et al. (59) (China)	Female SD rats (8 wk old, 200–250 g)	Impactor: 10 g, 5 cm, 10 s	15/15/15/15	A. SCI B. SCI + BMSC C. SCI + BMSC + EPO D. SCI + derive BMSCs for culture	5,000 UI/kg, *i.p.*, after injury, first 3 d	BBB scale, grid walk test of hind limbs
Ozkunt et al. (60) (Turkey)	Adult female SD rats (200–220 g)	Aneurysm clip: T9, 45 mm, 1 min	10/10/10	A. SCI B. SCI + MPSS C. SCI + EPO	5,000 UI/kg, intrathecal administration, after injury	BBB scale
Zhang et al. (61) (China)	Adult female SD rats (220–250 g)	Impactor: T10, 10 g, 2.5 cm	18/18/18/18	A. Sham B. Sham + rhEPO C. SCI D. SCI + rhEPO	5,000 UI/kg, *i.p*. after injury, 7 d	BBB scale
Wang et al. (62) (China)	Adult male SD rats (200–220 g)	Bulldog Clamp: T7-T10, 30 g force, 1 min	5/5/5/5	A. Sham + saline B. Sham + EPO C. SCI + saline D. SCI + EPO	2,000 UI/kg, i.p., after injury and 24 h	BBB scale, inclined plane test
Barros et al. (63) (Brazil)	Adult male Wistar rats (340–450 g)	Impactor: T10, 10 g, 12.5 mm	10/10/10/10/10	A. Sham B. SCI + placebo C. SCI + EPO D. SCI + EPO + IL-6 E. SCI + IL-6	1,000 UI/kg, *i.p*., after ischemia, 3 wk	BBB scale
Li et al. (64) (China)	Adult male SD rats (200–240 g)	-	20/20/20/20	A. SCI B. SCI + EPO + BDNF/BMSC C. SCI + EPO D. SCI + BDNF/BMSC	5,000 UI/kg, *i.p*., before injury	BBB scale
Zhong et al. (65) (China)	Adult SD rats (250–300 g)	Impactor: T10, 10 g, 5 cm	5/5/5/5	A. Sham + saline B. SCI + saline C. SCI + EPO (1,000 UI/kg) D. SCI + EPO (5,000 UI/kg)	1,000 UI/kg/ 5,000 UI/kg i.p., after injury and once a wk	BBB scale

*BBB scale, Basso, Beattie, and Bresnahan scale; EPO, erythropoietin; h, hour(s); i.p, intraperitoneally; MPSS, methylprednisolone sodium succinate; SCI, Spinal cord injury; SD, Sprague-Dawley; wk, week(s); min, minute(s); s, second; N, Newton; BDNF, brain-derived neurotrophic factor; BMSC, bone marrow stromal cell; IL-6, interleukin-6; NSCs, Neural stem cells; VEGF, vascular endothelial growth factor; FK 506, tacrolimus*.

In most studies EPO was administered to SCI rats immediately afterwards, either intraperitoneally or intravenously, at doses ranging from 100 to 5,000 UI/kg. The negative control was saline in most cases. All studies reported results by functional assessment or biochemical analysis.

### Bias analysis of included studies

The risks of bias for all 33 independent studies are shown in Table 2. The overall methodological quality of studies methodology is good. Thirty-three studies contained a statement of the peer-reviewed journal, blind established model, and anesthetics used without marked intrinsic neuroprotective properties. None of the studies have described whether animal models are affected by diabetes, advanced age, or high blood pressure. Ninteen studies described the allocation concealment and 17 studies described temperature control. Blinded assessment of the outcome was described in 15 studies. Thirty-two studies described the statement of compliance with animal welfare regulations and only two studies described calculation of sample size. Twenty-three studies contained a statement of potential conflicts of interest.

**Table 2:** Risk of bias in included studies accessed by CAMARADES.

**References**	**1**	**2**	**3**	**4**	**5**	**6**	**7**	**8**	**9**	**10**	**Score**
Gorio et al. (18)	+	+	+	+	?	+	?	?	+	?	6
Leist et al. (35)	+	?	?	+	?	+	?	?	+	?	4
Gorio et al. (36)	+	+	?	+	?	+	?	?	+	+	6
Grasso et al. (37)	+	+	?	+	?	+	?	?	+	-	4
Choi et al. (38)	+	?	?	+	+	+	?	?	+	?	5
Okutan et al. (39)	+	+	+	+	+	+	?	?	+	?	7
Vitellaro-Zuccarello et al. (40)	+	?	+	+	?	+	?	?	+	+	6
Fumagalli et al. (43)	+	+	?	+	?	+	?	?	+	?	5
Mann et al. (44)	+	+	+	+	+	+	?	?	+	+	8
Pinzon et al. (45)	+	+	?	+	?	+	?	?	+	?	5
Vitellaro-Zuccarello et al. (41)	+	?	+	+	+	+	?	?	+	+	7
Yazihan et al. (42)	+	+	?	+	?	+	?	?	+	?	5
Fang et al. (46)	+	+	+	+	?	+	?	?	+	+	7
Huang et al. (48)	+	+	+	+	?	+	?	?	+	+	7
Kontogeorgakos et al. (47)	+	?	+	+	+	+	?	?	+	?	6
Hong et al. (49)	+	?	+	+	?	+	?	?	+	+	6
Hong et al. (51)	+	?	+	+	?	+	?	?	+	+	6
Jin et al. (52)	+	?	?	+	+	+	?	?	+	+	6
Freitag et al. (53)	+	+	?	+	+	+	?	?	+	+	7
Wu et al. (55)	+	?	?	+	?	+	?	?	+	+	5
Yang et al. (54)	+	+	+	+	+	+	?	?	+	+	8
de Mesquita et al. (25)	+	+	?	+	+	+	?	+	+	+	8
Marcon et al. (56)	+	?	+	+	+	+	?	?	+	+	7
Kökoglu et al. (58)	+	?	+	+	?	+	?	?	+	+	6
Zhao et al. (57)	+	?	?	+	?	+	?	?	+	?	4
Hwang et al. (50)	+	+	+	+	+	+	?	?	+	+	8
Li et al. (59)	+	?	+	+	?	+	?	?	+	+	6
Ozkunt et al. (60)	+	?	+	+	?	+	?	?	+	+	6
Zhang et al. (61)	+	+	+	+	+	+	?	?	+	+	8
Wang et al. (62)	+	?	?	+	+	+	?	?	+	+	6
Barros et al. (63)	+	+	+	+	+	+	?	+	?	+	8
Li et al. (64)	+	?	+	+	?	+	?	?	+	+	6
Zhong et al. (65)	+	+	?	+	+	+	?	?	+	+	7

### The effects of EPO intervention on BBB score in rats with SCI

**Figure 2:**
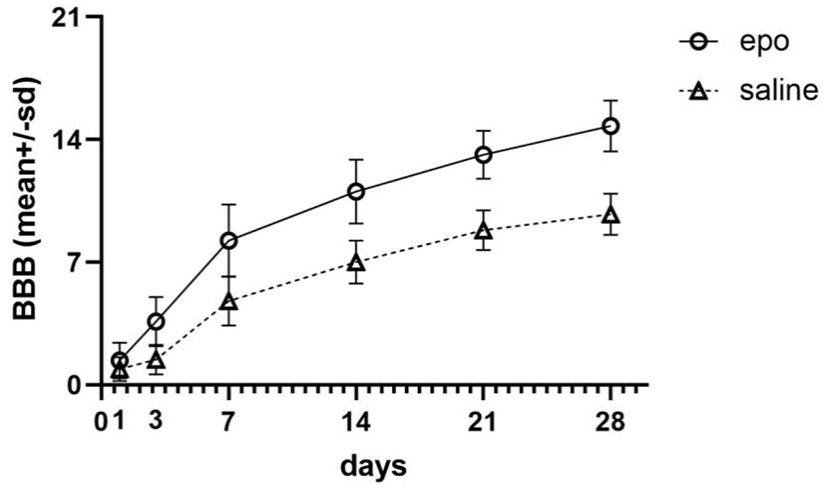
Evolution of the scores on the BBB of the two groups including the six (1, 3, 7, 14, 21, and 28 days) evolution periods. BBB, Basso-Beattie-Bresnahan locomotor rating.

The meta-analysis indicated that SCI rats receiving EPO therapy showed a significant locomotor function recovery in all studies (1 d to 28 d, *P* < 0.01; Figure 2). After the intervention of EPO, the BBBs score increased [1 d, 21 studies (18, 25, 35–37, 41, 43–46, 48, 49, 51, 54, 59, 61, 63–65), WMD = 0.69, 95% CI [0.22, 1.15], *P* = 0.004; 3 d, 17 studies (18, 25, 35, 42, 43, 46, 48, 49, 58, 63, 66–70), WMD = 2.50, 95% CI [1.69, 3.31], *P* < 0.00001; 7 d, 24 studies (18, 25, 35, 36, 42, 43, 45, 46, 48, 49, 54, 58, 59, 63, 65, 67–69, 71–74), WMD = 3.31, 95% CI [2.21, 4.41], *P* < 0.00001; 14 d, 20 studies (18, 25, 35, 42, 43, 45, 46, 48, 49, 58, 59, 63, 65, 67–69, 73, 74), WMD = 3.80, 95% CI [2.50, 5.09], *P* < 0.00001; 21 d, 18 studies (18, 25, 35, 42, 43, 45, 46, 48, 49, 58, 59, 63, 65, 67, 68, 73), WMD = 4.06, 95% CI [2.89, 5.88], *P* < 0.00001; 28 d, 19 studies (18, 25, 35, 37, 42, 43, 45, 46, 49, 58, 59, 63, 65, 67, 68, 73, 75), WMD = 4.88, 95% CI [3.57, 6.18], *P* < 0.00001;] in a random-effects model. The meta-analysis indicated that SCI rats receiving EPO therapy showed a significant locomotor function recovery after 14 days compared with control, then the superiority of EPO therapy maintained to 28 days (Figure 2).

Subgroup analysis was carried out according to the different animal models and species, as well as the different EPO administration. While, there was no difference between different animal models and species, then the subgroup analysis recommended 5,000 UI/kg is the more effective dose [7 d, 11 studies (18, 35, 36, 41, 44, 45, 54, 59, 61, 64, 65), WMD = 4.05, 95% CI (2.23, 5.88), *P* < 0.0001; Table 3], although its effect was not statistically different from that of 1,000 UI/kg [7 d, nine studies (18, 25, 43, 45, 46, 48, 49, 51, 63), WMD = 3.13, 95% CI (1.05, 5.21), *P* = 0.003; Table 3]. Then, the different administration methods of animals, single or multiple administration after SCI dis not affect the neuroprotective effect of EPO for SCI (Table 3).

**Table 3:** Subgroup analysis of BBB score.

**Pooled estimates (7 d)**	**No. of studies**	**WMD (95% CI)**	***P*** **-value**
**Model**			
Impactor	18	3.60 [2.73, 4.46]	*P* <0.00001
Aneurysm clip	7	2.16 [0.83, 3.49]	*P* = 0.002
**Species**			
SD rats	18	3.49 [2.20, 4.78]	*P* <0.00001
Wistar rats	5	1.77 [0.27, 3.28]	*P* = 0.02
**Dosage**			
1,000 UI/kg	9	3.13 [1.05, 5.21]	*P* = 0.003
5,000 UI/kg	11	4.05 [2.23, 5.88]	*P* <0.0001
**Administration time**			
Before modeling	2	0.91 [−1.01, 2.83]	*P*=0.35
After modeling	22	3.51 [2.59, 4.44]	*P* <0.00001
**After modeling**			
Once	13	3.30 [2.25, 4.35]	*P* <0.00001
Multi	9	3.88 [2.07, 5.68]	*P* <0.0001
**Mode of administration**			
Once	15	2.96 [1.50, 4.42]	*P* <0.00001
Multi	9	3.88 [2.07, 5.68]	*P* <0.00001
**Once**			
Before modeling	2	0.91 [−1.01, 2.83]	*P* = 0.35
After modeling	14	3.65 [2.62, 4.68]	*P* <0.00001
**Multi**			
>3 times	4	4.27 [1.61, 6.92]	*P* = 0.002
≤ 3 times, >1 time	5	3.28 [1.24, 5.32]	*P* = 0.002

*BBB, Basso-Beattie-Bresnahan locomotor rating; WMD, weighted mean difference; SD, Sprague-Dawley*.

### The effects of EPO intervention on cavity area and spared area in rats with SCI

**Figure 3:**
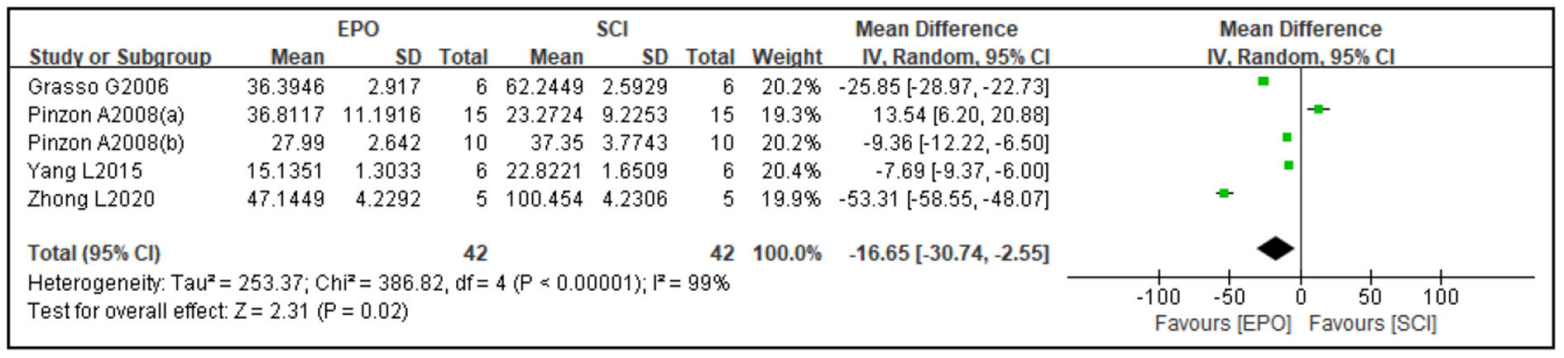
Forest plot for the effects of EPO intervention on cavity area in rats with SCI. EPO, erythropoietin; SCI, spinal cord injury.

**Figure 4:**
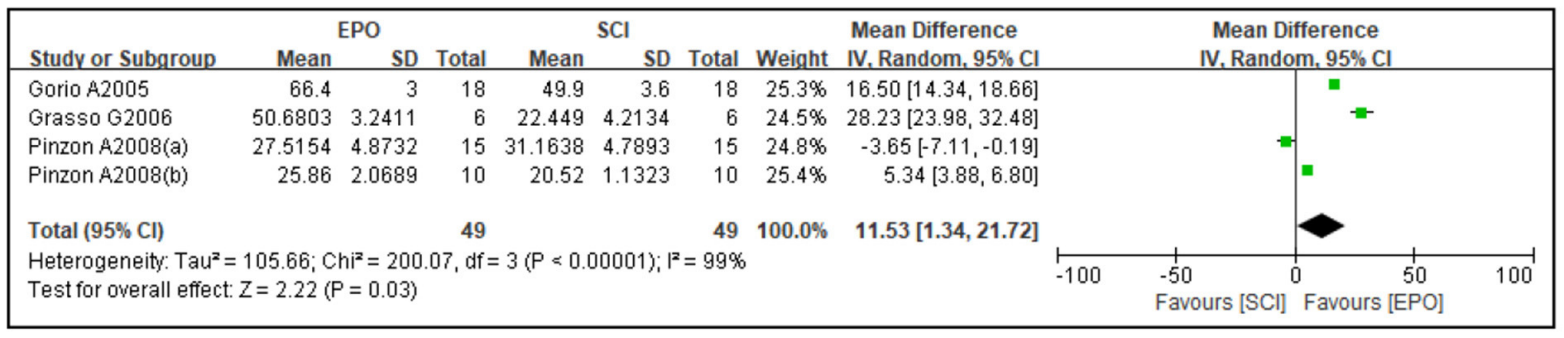
Forest plot for the effects of EPO intervention on spared area in rats with SCI. EPO, erythropoietin; SCI, spinal cord injury.

Four studies reported cavity area and three studies reported spared area as an outcome. Compared with the control group, the cavity area was reduced [four studies (37, 45, 54, 65), WMD = −16.65, 95% CI (−30.74 to −2.55), *P* = 0.02; Figure 3] and spared area was increased by EPO [three studies (36, 37, 45), WMD = 11.53, 95% CI (1.34 to 21.72), *P* = 0.03; Figure 4].

### The effects of EPO intervention on MDA in rats with SCI

**Figure 5:**
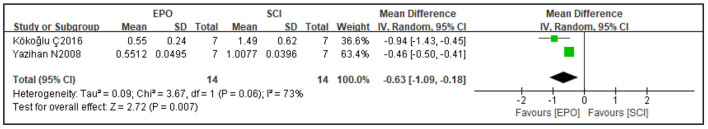
Forest plot for the effects of EPO intervention on MDA in rats with SCI. EPO, erythropoietin; MDA, malondialdehyde.

Antioxidative effects were measured in three studies. MDA has been widely used as a convenient biomarker for lipid peroxidation of omega-3 and omega-6 fatty acids due to its easy reaction with thiobarbituric acid (66, 67). Three studies measured MDA levels after SCI and found that MDA levels were significantly lower in the EPO group than the matched group of controls [2 studies (42, 58), WMD = −0.63, 95% CI (−1.09 to −0.18), *P* = 0.007; Figure 5] to detect its antioxidative effect.

### Publication bias

**Figure 6:**
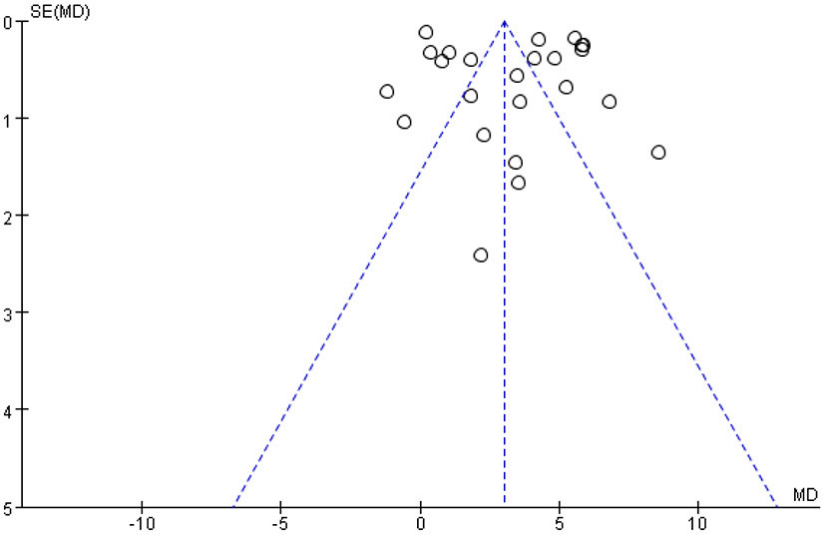
Funnel plots of publication bias for BBB score. BBB, Basso-Beattie-Bresnahan locomotor rating.

The funnel plot of the BBB score was essentially symmetrical, suggesting a low risk of publication bias (Figure 6).

## Discussion

### Summary of main results

Currently, this is the first meta-analysis of animal experiments where EPO improves neurological outcomes after SCI. A total of 33 studies were included. The overall methodological quality of the studies were rated as “good.” Twenty-five of 33 studies scored over six after quality assessment, which meant over 75% researches were advanced in methodology. Results showed that EPO had been closely associated with improving BBB score and spared area, as well as decreasing cavity area and MDA after injury *in vivo* animal studies, which indicated its inducing neurological recovery and antioxidant effects. Meanwhile, subgroup analysis showed that a single injection of 5,000 UI/kg after injury promoted the recovery of spinal cord function. Meanwhile, the differences of species, animal models and administration of EPO were insignificant on the neuroprotective effect of EPO against SCI. Moreover, the funnel plot is essentially symmetrical while the accuracy of the measurement results is robust.

### The possible mechanism for the effect of EPO in SCI

**Figure 7:**
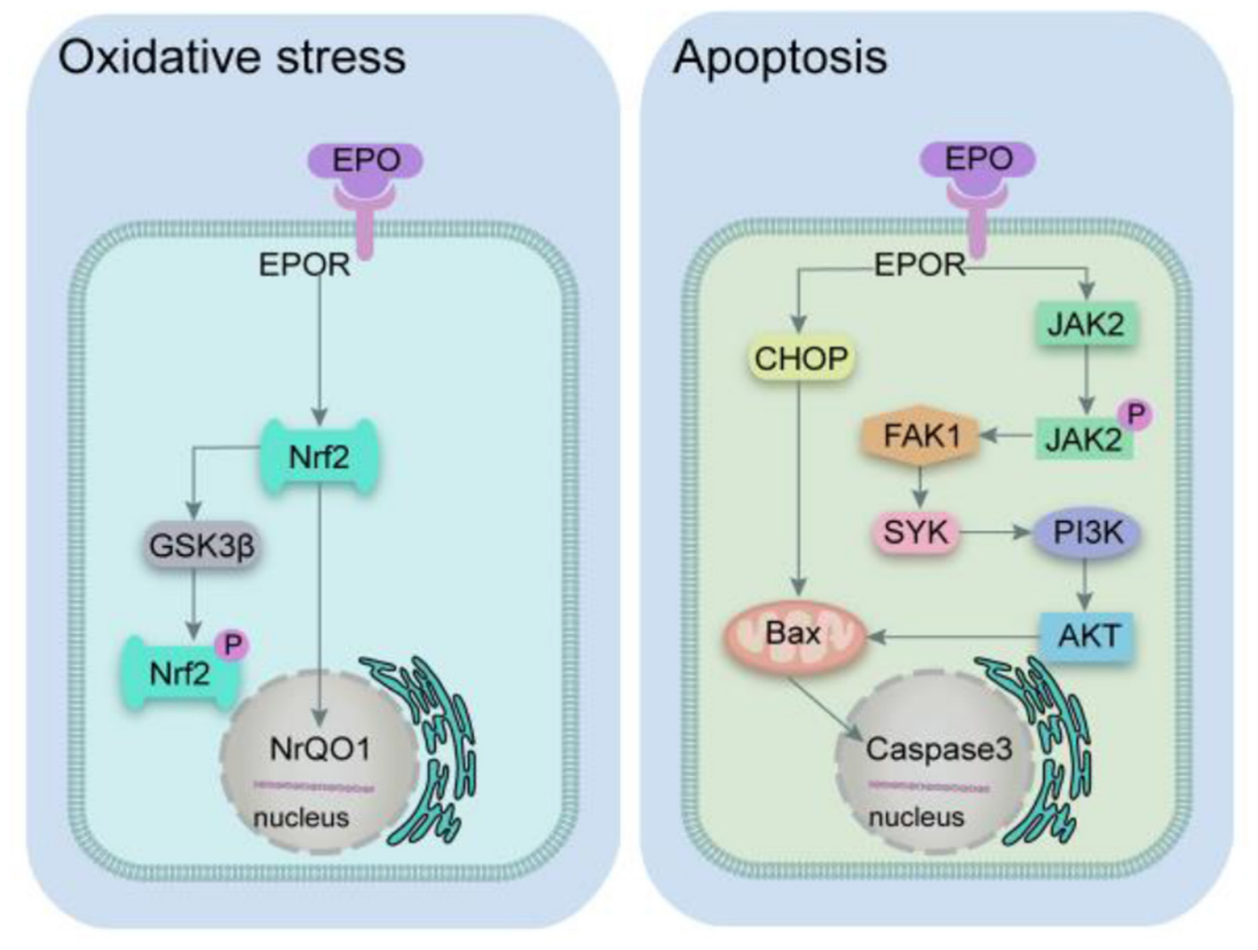
Mechanism diagram of EPO pathway. EPO, erythropoietin; EPOR, erythropoietin receptor; Nrf2, nuclear factor 2; GSK3β, glycogen synthase kinase 3 beta; NQO1, (NAD(P)H quinone dehydrogenase 1; CHOP, C/Ebp-homologous protein; BAX, Bcl2-associated x protein; JAK2, janus activated kinase 2; FAK1, focal adhesion kinase 1; SYK, spleen tyrosine kinase; PI3K, phosphatidylinositol 3-kinase; AKT, AKT serine/threonine kinase.

According to the pathology of SCI, it can be categorized into primary and secondary SCI. Primary injury is caused mainly by mechanical damage resulting in the destruction of the local neural tissue. Secondary injury develops gradually on the basis of the primary injury and includes oxidative stress, apoptosis, and autophagy (68–73). The potential mechanism for the effect of EPO in SCI is summarized in Figure 7 and Table 4.

**Table 4:** The signal pathway of EPO for SCI.

**References**	**Mechanism**	**Signal pathway**
Gorio et al. (18)	Apoptosis, Inflammation	/
Leist et al. (35)	Apoptosis	STAT-5, JAK2, IL6, MCP-1
Gorio et al. (36)	Inflammation	MIP-2, IL-8, TNF-α, IL-6, IL-1β
Grasso et al. (37)	Inflammation	GFAP
Choi et al. (38)	Apoptosis	GFAP, VEGF, MAP-2
Okutan et al. (author?) (39)	Apoptosis	caspase-3, MPO
Vitellaro-Zuccarello et al. (40)	Inflammation	NG2, GFAP, CNPase, ED1, 5HT, CSPG
Vitellaro-Zuccarello et al. (41)	Oxidative stress	AQP4, GFAP, CSPG, vimentin, dystrophin, syntrophin
Yazihan et al. (42)	Apoptosis, Oxidative stress	GSH, MDA, CAT, TNF-α
Fumagalli et al. (43)	Apoptosis	NGF, BDNF, FGF-2, CNTF, GDNF
Mann et al. (44)	Apoptosis	/
Pinzon et al. (45)	Apoptosis	/
Fang et al. (46)	Oxidative stress	TSP-1, TGF-β
Kontogeorgakos et al. (47)	Apoptosis	/
Huang et al. (48)	Apoptosis, ER stress	p-ERK, MKP-1
Hong et al. (49)	Apoptosis	PDGF
Hwang et al. (50)	Apoptosis	MDI
Hong et al. (51)	Apoptosis	CHOP
Yilmaz (2012)	Apoptosis	MDA, Caspase-3
Jin et al. (52)	Oxidative stress	Nrf2, NQO1, GST
Freitag et al. (53)	Apoptosis	
Yang et al. (54)	Apoptosis, Oxidative stress	CD68, LFB
Simon et al. (76)	Apoptosis	Chitinase 1, Chitinase 3, Stathmin, EF1α, senescence
Wu et al. (55)	Apoptosis	Bcl-2, Caspase-3
de Mesquita et al. (25)	Apoptosis	/
Marcon et al. (56)	Apoptosis	/
Zhao et al. (57)	Apoptosis	ADM
Kökoglu et al. (58)	Oxidative stress	MDA, TAOC
Li et al. (59)	Apoptosis	VEGF, BDNF
Ozkunt et al. (60)	Apoptosis	PDGF-β, GFAP
Zhang et al. (61)	Apoptosis	BrdU, β-tubulin, GFAP, TRITC, FITC
Wang et al. (62)	Autophagy	p-AMPK/AMPK, p-mTOR/mTOR, p-p70S6K/p70S6K, LC3-I, LC3-II, Beclin, p62
Barros et al. (63)	Apoptosis, Inflammation	IL-6
Li et al. (64)	Apoptosis	BDNF
Zhong et al. (65)	Autophagy, ER stress	ERK/p-ERK, p-mTOR/mTOR, LC3 A/B, Beclin1, p62, GAP43, CD86, TNF-α, iNOS

*5HT, 5-hydroxytryptamine; ADM, adrenomedullin; AMPK, adenosine 5'-monophosphate (AMP)-activated protein kinase; AQP4, aquaporin 4; Bcl-2, b cell lymphoma 2; BDNF, brain-derived neurotrophic factor; BMSC, bone marrow stromal cell; BrdU, 5-bromo-2'-deoxyuridine; CAT, catalase; CHOP, C/Ebp-homologous Protein; CNPase, 2',3'-cyclic nucleotide 3'-phosphodiesterase; CNTF, ciliary neurotrophic factor; CSPG, chondroitin sulfate proteoglycan; EF1α, elongation factor 1-alpha; ERK, extracellular regulated protein kinases; FGF-2, fibroblast growth factor-2; FITC, fluorescein isothiocyanate; GAP43, growth associated protein-43; GDNF, glial cell derived neurotrophie factor; GFAP, glial fibrillary acidic protein; GSH, glutathione; GST, glutathione s-transferase; IL-1β, interleukin-1β; IL-6, interleukin-6; IL-8, Interleukin-8; iNOS, inducible nitric oxide synthase; JAK2, janus kinase 2; LC3, light chain 3; MAP-2, microtubule Associated Protein 2; MCP-1, monocyte chemotactic protein 1; MDA, malondialdehyde; MIP-2, macrophage inflammatory protein-2; MKP-1, mitogen-activated protein kinase; MPO, myeloperoxidase; mTOR, mammalian target of rapamycin; NGF, nerve growth factor; NQO1, nitroquinoline oxide 1; Nrf2, nuclear factor 2; p-AMPK, phospho-AMPK; PDGF, platelet derived growth factor; p-ERK, PKR-like endoplasmic reticulum kinase; p-mTOR, phospho-mTOR; p-p70S6K, phospho-p70 S6 kinase; STAT-5, signal transducers and activators of transcription 5; TAOC, tactical air operations center; TGF-β, transforming growth factor-β; TNF-α, tumor necrosis factor-α; TRITC, Tetramethylrhodamine; TSP-1, thrombin-sensitive protein; VEGF, vascular endothelial growth factor*.

#### Protective effect of EPO related to oxidative stress

SCI is mainly a physical injury, which releases a lot of reactive oxygen species (ROS) and causes oxidative stress reactions in cells. It is widely believed that oxidative stress injury causes cell and tissue damage after SCI. One of the mechanisms of EPO treating SCI is inhibiting oxidative toxicity of cells (74, 75). This study showed that EPO against SCI mainly attributed to reducing MDA levels which was an important indicator of oxidative stress. Recent studies have demonstrated that nuclear factor 2 (Nrf2) is the core transcription factor of antioxidant response of exogenous stimuli. Under the conditions of oxidative stress, Nrf2 goes through the cytoplasm and enters the nucleus, which regulates the expression of cytoprotective enzymes such as nicotinamide adenine dinucleotide phosphate quinone oxidoreductase 1 (NrQO1) and glutathione s-transferase (GST), thus reducing cell oxidation and inflammation (77). Oxidative stress causes endoplasmic reticulum (ER) stress which disrupts protective signals thus leading to glycogen synthase kinase 3 beta (GSK-3β) phosphorylation and increasing in mitochondrial GSK-3β. This dual mechanism aggravates ER stress inhibiting EPO-induced suppression (78). The exogenous administration of recombinant EPO can activate Nrf2 signal pathway. Therefore, it was suspected that EPO inhibits oxidative stress through the classical Nrf2 signaling pathway in the SCI model.

#### Protective effect of EPO related to apoptosis

The C/Ebp-homologous Protein (CHOP) is a symbolic gene of endoplasmic reticulum stress-induced apoptosis, and cells involved in endoplasmic reticulum stress-regulated apoptosis and deficient expression of CHOP gene significantly reduce cell death induced by endoplasmic reticulum stress. Continuous activation of CHOP can down-regulate the anti-apoptotic protein b cell lymphoma 2 (Bcl-2), and then activate the mitochondrial apoptosis pathway regulated by Bcl2-associated x protein (Bax) (79). The anti-apoptotic mechanisms of phosphatidylinositol 3-kinase/serine/threonine kinase (PI3K/Akt) signaling pathway are mainly due to the direct regulation of Bad kinase, the transcription factor Forkhead, and the inhibition of mitochondrial apoptosis factor release (80–83). SCI leads to local inflammatory reaction and releases a variety of cytokines, which can regulate cell apoptosis by activating janus activated kinase 2/signal transducer and activator of transcription (JAK-STAT) signaling pathway (84). STAT3 inhibits neuronal apoptosis by inducing the expression of genes related to cell growth and repair, such as Bcl-2 and Bcl-xl (85). However, STAT1 played a role in vinegar cell apoptosis by inhibiting the expressions of Bcl-2 and Bcl-xl and up-regulating the expressions of caspase-1, caspase-2 and caspase-3 (86–88). Exogenous administration of EPO can reduce tissue damage and apoptosis by improving the expression of anti-apoptotic genes and telomerase activity (89). EPO can activate PI3K/Akt, JAK/STAT signaling pathways and improve ERS, thus enhancing cell repair capacity and resisting apoptosis (90–92).

### Strengths and limitation of evidence

In this study, the advantages of systematic and experimental evaluation were combined, and the summarized data indicated that EPO was a promising drug for the recovery of neurological function. Meanwhile the action mechanism of EPO was summarized. An in-depth understanding of the mechanism of EPO treatment for SCI will not only contribute to the exploration of the pathological mechanism of SCI, but also was conductive to exploring new and effective drugs for further development and related transformation research. Among the limitations of the evidence, MDA and spared area only have two to four studies, with relatively low evidence strength. Rats of other ages or other kinds of experimental animals lack a certain quantity and quality of research, so comprehensive evidence cannot be provided. Inevitably, this systematic review has some limitations. First, since we were unable to obtain data on individual animals, we could only conduct a meta-analysis of the overall level of each study. Although we used the standardized mean difference to reduce the length of the statistical effect, we did not completely eliminate the bias, which is also an important factor affecting the quality of the study. Different SCI models were used in each study, for example, administration method (single or multiple administration). A simple hybrid approach is not the most appropriate approach, but we cannot layer-analyze all inconsistencies.

## Conclusions

Based on the results of this meta-analysis, we demonstrated that EPO intervention could improve neurological recovery and antioxidative effects in rat models of SCI. Our results show that the mechanism of EPO preventing neuronal apoptosis is mainly through the alleviation of oxidative stress. The results of this meta-analysis must be interpreted and applied with an appropriate degree of caution because some factors such as the failure to use more novel research methods may overestimate the efficacy of treatment. Even so, EPO could be a promising drug to treat SCI. In the future, it deserves a larger study that takes into account the design features recommended in the discussion of this meta-analysis.

## Data availability statement

The original contributions presented in the study are included in the article/supplementary material, further inquiries can be directed to the corresponding authors.

## Author contributions

Y-yZ and MY designed the study and did the data analysis. KZ and R-rX collected the data. Y-yZ, MY, and J-hX wrote the manuscript. X-jC and WM revised the manuscript and decided to submit the manuscript for publication. All authors contributed to the article and approved the submitted version.

## Funding

The financial support for this work are provided by the Research Project of Shanghai Science and Technology Commission (19401901100 and 21S21900500), Shanghai Municipal Health Commission (No. 2021LPTD-008), The Fifth Batch of Scholars of Longhua Hospital Affiliated to Shanghai University of TCM (KC2022006), The program for Longhua Medical Scholar (No. YM2021007), National Natural Science Foundation of China (No. 82174409 for MY and No. 82074454 for X-jC), China Association of Traditional Chinese Medicine Youth Talent Promotion Project (No. CACM-2021-QNRC2-B23 for MY), and Shanghai Natural Science Foundation (No. 20ZR1459000 for MY and 22ZR1461700 for X-jC).

## Conflict of interest

The authors declare that the research was conducted in the absence of any commercial or financial relationships that could be construed as a potential conflict of interest.

## Publisher's note

All claims expressed in this article are solely those of the authors and do not necessarily represent those of their affiliated organizations, or those of the publisher, the editors and the reviewers. Any product that may be evaluated in this article, or claim that may be made by its manufacturer, is not guaranteed or endorsed by the publisher.
